# Editorial for the Special Issue on Tunable Nanophotonics and Reconfigurable Metadevices

**DOI:** 10.3390/mi14030544

**Published:** 2023-02-26

**Authors:** Yuancheng Fan, Benfeng Bai, Yusheng Lin

**Affiliations:** 1 School of Physical Science and Technology, Northwestern Polytechnical University, Xi’an 710129, China; 2 Department of Precision Instrument, Tsinghua University, Beijing 100084, China; 3 School of Electronics and Information Technology, Sun Yat-sen University, Guangzhou 510006, China

Photonic nano/microstructures (e.g., metamaterials and metasurfaces) with tunable properties provide new possibilities for the manipulation of light–matter interactions at the nanoscale, enabling many emerging technologies for novel applications, such as advanced imaging, large-bandwidth and high-speed communications, flexible and high-contrast displays, high-capacity data storage, optical computing, and biomedical technologies. Tunable nanophotonics and reconfigurable metadevices are becoming hotspots in optics as the precise control of optical properties becomes more possible and future applications are discovered. The implantation of various tunable materials or reconfigurable structures is a straightforward method for the realization of tunable nanophotonics. Recently, an increasing number of studies have paid attention to active tuning and driving methods in metamaterials/metasurfaces to increase their flexibility and applicability in nanophotonics. This Special Issue aims to collate contributions in the fields of tunable nanophotonics and reconfigurable metadevices.

This Special Issue, entitled “Tunable Nanophotonics and Reconfigurable Metadevices”, includes some remarkable studies: four research articles and one review article. The issue covers a wide range of topics: tunable localized surface plasmon resonance (LSPR) in Ag nanobars [1], electrically tunable lithium niobate on an insulator (LNOI) metasurface for on-chip optical beam control [2], on-chip metasurface-based quantum analog devices [3], a bifunctional reconfigurable metalens made from phase-change materials [4], and a review of recent advances in optical metasurface research [5]. From the perspective of basic optical properties, Wu et al. [1] reported the study of LSPR in synthesized large-size Ag nanobars (length: 400~1360 nm) working in a longer near-infrared wavelength range (>1000 nm). The LSPR in an Ag nanobar can be flexibly tuned in a wide wavelength range (400~2000 nm) by changing the geometry of the nanostructure. From the perspective of optical functionality, Dou et al. [2] propose an electrically tunable LNOI metasurface for on-chip optical beam manipulation, where dynamic focusing function and deflection function are demonstrated on a metasurface with varying voltages, considering the electro-optic effect of lithium niobate. Wei et al. [3] proposed an on-chip quantum searcher based on a silicon-on-insulator (SOI) structure, which comprises four on-chip metasurfaces with controlled transmitted intensity and phase profiles for simulating a quantum search algorithm. A reconfigurable metalens made from phase-change materials is presented by Ma et al. [4] for use as a bifunctional response to 3D depth imaging. Finally, in their review, Ullah et al. [5] discuss three fundamental mechanisms of wavefront modulation for metasurfaces and research advances regarding metasurfaces in the fields of wavefront modulation and holographic displays, as well as addressing novel research directions, such as metalens devices, cascaded metasystems, and vortex beam generation.

Overall, this Special Issue provides an interesting flavor of the diversity of tunable nanophotonics and metadevices incorporated with tunable or reconfigurable mechanisms. These new results demonstrate the most recent advances in tunable metamaterials and their potential usefulness in various aspects, both experimentally and theoretically. We expect that more and more tunable and reconfigurable metasurfaces/optical nanostructures with smart properties will emerge in the future for functional metadevices.
